# Acknowledgement to Reviewers of *Tropical Medicine and Infectious Disease* in 2018

**DOI:** 10.3390/tropicalmed4010009

**Published:** 2019-01-08

**Authors:** TropicalMed Editorial Office

**Affiliations:** MDPI, St. Alban-Anlage 66, 4052 Basel, Switzerland

Rigorous peer-review is the corner-stone of high-quality academic publishing. The editorial team greatly appreciates the reviewers who contributed their knowledge and expertise to the journal’s editorial process over the past 12 months. In 2018, a total of 126 papers were published in the journal, with a median time to first decision of 14 days and a median time to publication of 35.5 days. The editors would like to express their sincere gratitude to the following reviewers for their cooperation and dedication in 2018:

Abanyie, Francisca A.Lewin, MattAinsworth, StuartLi, YingAlfson, KendraLidbury, BrettAllavena, RachelLodh, NilanjanAllou, NicolasLouis, Valérie R.Alspaugh, J. AndrewLuce-Fedrow, AlisonAmemiya, KeiLukšić, BorisAngheben, AndreaMajumdar, SumanArrey, Agnes EbotabeMarks, MichaelArthurs, BryanMars, MauriceAvilés, Marta MartínezMartinez, JuanAye, KhinMasanotti, GiuseppeBaca, ArthurMaslow, Joel N.Badu, EmmanuelMason, PaulBaird, Robert W.Mauldin, Matthew R.Bajer, AnnaMcBride, Jere W.Baker, AnthonyMcBride, JohnBallardini, MarcoMcConkey, SandraBangs, JamesMcVey, David ScottBarbet, AnthonyMeggs, William J.Barkham, TimothyMelrose, WayneBartoloni, AlessandroMendoza-Cano, OliverBasu, PratyushaMieras, LiesbethBecher, HeikoMiguel, SilviaBelo, SilvanaMitre, EdwardBernardo, UmbertoMlocicki, DanielBerthold-Pluta, AnnaModahl, CassandraBertoni, GiuseppeMokili, JohnBeyhan, SinemMonchatre-Leroy, ElodieBhave, DevayaniMone, HélèneBilliald, PhilippeMorici, LisaBisoffi, ZenoMoro, Pedro L.Blacksell, StuartMotaze, VillyenBlanton, LucasMurdoch, Michele E.Blasdell, KimMusto, JennieBlok, David J.Mutinelli, FrancoBlumberg, LucilleNaafs, BernardBorlee, BradNaber, KurtBoufana, BelgeesNading, AlexanderBowen, RichardNagata, ToshiBowman, DwightNakazawa, YoshinoriBoyton, Rosemary J.Natale, AldaBradbury, RichardNdione, Jacques-AndréBraks, MarietaNery, Susana VazBrough, MarkNeubauer, HeinrichBrown, Catherine M.Nichols, GordonBudge, Philip J.Nielsen, Vance G.Burg, GunterNishizono, AkiraButler, ThomasOhta, NobuoCalvete, Juan J.Ortu, GiuseppinaCaprari, PatriziaOttesen, EricCarr, Silvana B.Owino, Simon OderaCarroll, RachelPakharukova, MariyaCentis, RosellaPark, JulieChambers, ThomasPekrun, ArnulfChami, GoylettePentakota, Sri RamChang, AileenPerdomo, DorandaChao, Chien-ChungPeto, Thomas J.Chaves, Luis FernandoRaby, EdwardChen, Ya-LeiRahman, Mohammed S.Chen, Kow-TongRajamani, SathishChiou, Chien-ShunRamaswamy, KalyanasundaramChippaux, Jean-PhilippeRe, FabioChochlakis, DimosthenisResiere, DaborCissé, GuéladioRicca, EzioClark, Nicholas J.Ripoll, Fabienne NeulatCoelho, Ana CláudiaRockman, StevenCoertse, JessicaRodriguez-Andres, JulioColebunders, RobertRollinson, DavidConger, NicholasRosa, Bruce A.Conn, David BruceRoss, Kirstin E.Corea, EnokaRoy, ChadCork, SusanRuktanonchai, Nick WarrenCrellen, ThomasRupprecht, CharlesCroll, RogerRusso Frasca Candido, RenataCronin, AidanSaez, MarcCrouch, AlanSalje, JeanneCurrie, BartSalvador, FernandoCutler, Sally JaneSando, EiichiroDaley, PeterSangare, LauraDarbro, JonathanSarkar-Tyson, MitaliDe Figueiredo, PaulSarovich, Derek S.De Haro, LucSato, Marcello OtakeDe Miguel, SilviaSaturnino, CarmelaDe Paula, Fabiana MartinsScavuzzo, Carlos M.DeShazer, DavidSchaer, MichaelDey, AditiSchaer, JulianeDkhar, Hedwin KitdorlangSchempp, Christoph M.Dobos, Karen M.Schroeder, BetsyDouglass, JanetSchully, Kevin L.Dreassi, EmanuelaScotti, MarcusDuffy, TomasSecor, William EvanDunachie, Susanna J.Segal, RinaDuthie, Malcolm S.Sengupta, Mita EvaDyson, LouiseShaw, MarcEbbs, Erika T.Shelite, Thomas RobertEI Ridi, RashikaShimozako, Helio JunjiEkundayo, OlugbemigaSiddiqui, AfzalErokhin, VasiliiSingh, BrajendraFarrell, RobertSiqueira-Filha, NoemiaFeldman, Steven R.Šmajs, DavidFernandez-Prada, ChristopherSmiley, Sarah L.Fine, Paul EmSmith, SimonFischer, KatjaSmith, JessicaFisher, DaleSmith-Vaughan, HeidiFölster-Holst, ReginaSolís García Del Pozo, JuliánFranklin, PeterSpear, RobertFrentiu, FrancescaSpencer, John S.Galactionova, KatyaSprague, Lisa D.GAN, Yunn HwenSteinmetz, IvoGe, CuiStevens, Joanne M.Gee, JayStilianakis, NikolaosGiacani, LorenzoStolk, Wilma A.Gilchrist, CarolSuh, EunhoGillespie, JosephSulis, GiorgiaGnanathasan, Christeine AriaraneeTai, WanboGonzález Cappa, Stella MarisTan, Gek Yen GladysGordon, Catherine A.Tan Soo, Jie ShengGoto, KazuoTate, Rothwelle J.Grandahl, MariaTennessen, Jacob A.Grosset, JacquesTeo, AndrewHaas, WifriedThibault, FrançoisHalvorsen, PriyaThornley, SimonHamer, GabrielTilakaratne, DevHaque, UbydulTilley, ElizabethHarada, HidenoriTisch, Daniel J.Hasegawa, HideoToor, JaspreetHashem, Anwar M.Torina, AlessandraHayes, WilliamTrinh, TrungHead, MichaelTsai, Kun-HsienHeinzen, RobertTsai, Pei-JaneHeukelbach, JorgTsatsaris, AndreasHill, SimonTuanyok, ApichaiHoff, RodneyTuells, JoséHopkins, AdrianTurner, PaulHribar, LawrenceTurner, Hugo C.Huhtamo, EiliUmhang, GéraldIde, KazukiUtzinger, JeurgIwanicki, AdamVale, NunoJalava, KatriVan Brakel, WimJeong, Hye WonVan Der Werf, T.S. (Tjip)Jetybayev, Ilyas YerkinovichVan Lieshout, LisetteJones, Rodney P.Veraldi, StefanoJoseph, HayleyVincent, Richard L.Judd, JenniVitale, MariaKaranis, PanagiotisVogels, ChantalKaushik, AmitWagner, AbramKazimírová, MariaWalke, Henry T.Kearns, Therese M.Wang, Pa-ChunKeller, Peter M.Wangrangsimakul, TriKelly, DarylWarner, JeffreyKhatri, Vishal KishorWasko, DennisKling, KerstinWebster, BonnieKo, Wen-ChienWelkos, SusanKobayashi, YukiWest, T. EoinKoralur, MunegowdaWiener-Well, YonitKozubowski, LukaszWilson, R AlanKumar, NirbhayWingfield, TomKuo, Chi-ChienWongratanacheewin, SurasakLahondere, ChloeWu, HannahLamberton, PoppyWuthiekanun, VanapornLand, Kenneth C.Yagupsky, PabloLauridsen, Jørgen TrankjærYeh, Yin-TingLaustsen, Andreas HougaardYotsu, Rie RoselyneLaverack, GlennYoung, SeanLee, SunheeZhan, BinLeger, ElsaZhou, YibiaoLeonardo, Lydia R.Zienius, Dainius

